# Calcium wastes as an additive for a low calcium fly ash geopolymer

**DOI:** 10.1038/s41598-023-43586-w

**Published:** 2023-09-28

**Authors:** Prinya Chindaprasirt, Ubolluk Rattanasak

**Affiliations:** 1 Department of Civil Engineering, Faculty of Engineering, Sustainable Infrastructure Research and Development Center, Khon Kaen University, Khon Kaen, 40002 Thailand; 2 Academy of Science, Royal Society of Thailand, Dusit, Bangkok, 10300 Thailand; 3 Department of Chemistry, Faculty of Science, Burapha University, Chonburi, 20131 Thailand; 4 Center of Excellence on Environmental Health and Toxicology (EHT), OPS, MHESI, Bangkok, 10400 Thailand

**Keywords:** Civil engineering, Techniques and instrumentation

## Abstract

A geopolymer is a low-carbon cement based on the utilization of waste ash in alkali-activated conditions. Coal fly ash is widely used as a source material for geopolymer synthesis since it contains a sufficient amount of reactive alumina and silica for geopolymerization. Geopolymer products are known to have beneficial fire resistance and mechanical properties. Class F or low-calcium fly ash (LCFA) is generally used as a primary aluminosilicate source; however, heat curing is required to complete the reaction and hardening process and achieve a strong composite. Furthermore, calcium additives are often required to improve the strength of LCFA geopolymers. This paper presents the potential of reusing calcium waste for this purpose. Three calcium wastes, namely calcium carbide residue (CCR), limestone waste, and waste cement (WC) slurry in powder form were used as additives and compared with the use of ordinary Portland cement (OPC). LCFA was replaced with the calcium additives at 20%. However, 20% CCR resulted in flash setting, hence 5% CCR was used instead. A durability test using 3% HCl solution was also performed. The results showed that the reactivity of calcium additives played an important role in strength development. In the calcium–aluminosilicate–alkali system, calcium silicate hydrate (CSH) and calcium aluminosilicate hydrate (CASH) were formed. The maximum strength of 21.9 MPa was obtained from the OPC/LCFA geopolymer, and 3% HCl solution had a deleterious effect on the strength. OPC and CCR were favorable reactive sources of calcium compounds to blend with LCFA. From the thermogravimetric results, lower thermal weight changes with higher strength gains were achieved. Low CaCO_3_ decomposition at 750 °C according to the TGA curves indicated the more formation of thermally stable CSH and high compressive strength of Ca/LCFA geopolymers.

## Introduction

The construction industry consumes high quantities of Portland cement, and a ton of Portland cement production generates nearly a ton of CO_2_. In total, cement production accounts for 7% of the world’s greenhouse gas emissions^1^. In 2017, the world cement production reached 4100 million tons, resulting in the third-largest source of CO_2_ emissions^2^. It has been reported that a ton of cement production consumes 60–130 kg of fuel depending on the production process; the primary fuels used in the cement kiln are coal, petroleum coke, and natural gas^3,4^. Recently, green cements or low-carbon cements have been developed to reduce CO_2_ emissions and energy consumption in cement production. These cements include “pozzolan cement,” in which pozzolanic material is used to partially replace Portland cement, and “geopolymers,” which utilize silica- and alumina-rich materials to produce alkali-activated composites. However, further development of the composite properties is required to achieve environmentally friendly, strong, and durable products with an increased service life^5,6^.

Pozzolans are defined in ASTM C 618-23^7^ as siliceous or siliceous and aluminous materials that can react with calcium hydroxide to form cementitious compounds. Fly ash is a solid waste from coal-fired power plant and is extensively used as a pozzolan to partially replace cement because of its availability and high activity. Using fly ash can save several million dollars in the national economy^8^. However, the level of fly ash replacement is generally restricted to less than 40% of Portland cement^9^ In addition to pozzolan cement, geopolymers are another green binder, and various industrial and agricultural ashes can be used in their production.

A geopolymer is a type of alkali-activated material in which the aluminosilicate ashes are activated with an alkaline solution, and heat curing is often applied^6^. The leaching of amorphous phases of silica (SiO_2_) and alumina (Al_2_O_3_) in waste ashes in the presence of alkaline solutions results in the formation of an inorganic polymer chain. Coal fly ash is a widely used source material for geopolymer synthesis because it contains sufficient amounts of reactive alumina and silica for in situ geopolymerization reactions. The manufacturing of geopolymers involves lower CO_2_ emissions compared with conventional binders, and geopolymers exhibit advantageous acid and fire resistance and mechanical properties^10,11^. Thus, geopolymers are an alternative to Portland cement, and they have attracted a great deal of attention in the past 30 years.

Class F fly ash has been widely used as a primary aluminosilicate source of soluble Si and Al to synthesize geopolymers. When the rate of dissolution of Al from the fly ash is insufficient to produce a geopolymer gel, the addition of kaolinite is necessary. Heat curing is also required to achieve higher strength. Recently, Class C fly ash has received significant attention because it allows geopolymers to be cured at ambient temperature with sufficient strength^12^. The calcium content in Class C fly ash plays an important role in the strength development of geopolymers, owing to the coexistence of calcium silicate hydrate (CSH) in geopolymer products. Class C fly ash is available in Thailand as the byproduct of the largest lignite coal-fired power plant. Combustion of low-grade lignite results in a high calcium content of the fly ash, up to 30%^13^. The demand for this lignite fly ash in the construction sector is increasing, leading to an increase in the price. In comparison, Class F fly ash is also abundantly sourced from independent power producers using bituminous coal as a feedstock. An insignificant amount of this fly ash has been utilized because of a lack of published research, leading to low consumer confidence. Furthermore, researchers have only focused on the use of Class F fly ash in non-load-bearing geopolymers, including acidic wastewater treatment and CO_2_ capture materials^14,15^.

Several researchers have investigated the incorporation of calcium additives in the forms of calcium oxide, hydroxide, and carbonate to improve the overall strength^16–18^. A proper amount of calcium carbonate in the mixture governs the setting time and compressive strength according to the synergistic coexistence of CSH and the filler effect. Highly alkaline solutions also lead to the precipitation of calcium hydroxide, resulting in the increased solidification of the geopolymer^19^. Many industrial wastes have a high calcium content, such as calcium carbide residue (CCR), limestone waste, and waste cement (WC) slurry in powder form. These wastes contain a calcium content of more than 60% and require well-managed reuse and disposal. A portion of CCR can be used as supplementary cementing materials for cement and concrete^20,21^. However, recycling is limited considering the unsoundness of cement and concrete. Researchers have studied on CCR as a raw material for calcium carbonate (CaCO_3_) synthesis^22,23^, as an adsorbent to reduce the exhaust emissions of motorcycle^24^, and for soil stabilization^25^. Since, the annual global production of CCR is approximately 25 million tons in year 2021^26^, therefore, the utilization of CCR is needed.

In addition, washing the ready-mix concrete trucks generates concrete washing slurries in which coarse and fine aggregates are separated and recycled, leaving the cement paste powder. Then, thermal treatment at 800 °C for 3 h results in a reactive cementitious powder^27^. In limestone processing, limestone is crushed into coarse aggregate for the construction industry, and 20% of the production becomes limestone powder waste^28^. This limestone powder is generally disposed of in landfills, resulting in negative impacts on the environment and human lives.

Researchers have studied various calcium compounds in geopolymer production, particularly in high calcium fly ash geopolymer^29,30^. It has been reported that the strength of CCR/fly ash geopolymers increases with curing time owing to the co-existance of CSH with sodium aluminosilicate hydrate gels^29^. When OPC was used as an additive in high calcium fly ash geopolymer^30^, the final setting time was greatly reduced from 47 to 10 min for 10 M NaOH concentration. These materials are suitable for use as a repair binder. However, the utilization of these calcium sources has not been well studied in low calcium fly ash geopolymer. In addition, thermal study and elemental mapping of this geopolymer type have rarely been reported. Therefore, the utilization of WC and limestone powder waste in the geopolymer composites is very limited^31^. To utilize the industrial wastes, this work focused on the synergistic effect of various calcium wastes in Class F fly ash geopolymers. Low-temperature curing was applied, which can theoretically be obtained from outdoor heat exposure and power plant waste heat. Considering the addition of calcium compounds, the thermal behavior and durability of the geopolymers, as well as the microstructure and morphology, were reported. The results may serve as guidelines for the selection of calcium-containing waste in Class F fly ash geopolymers.

## Materials and methods

### Materials

**Figure 1:**
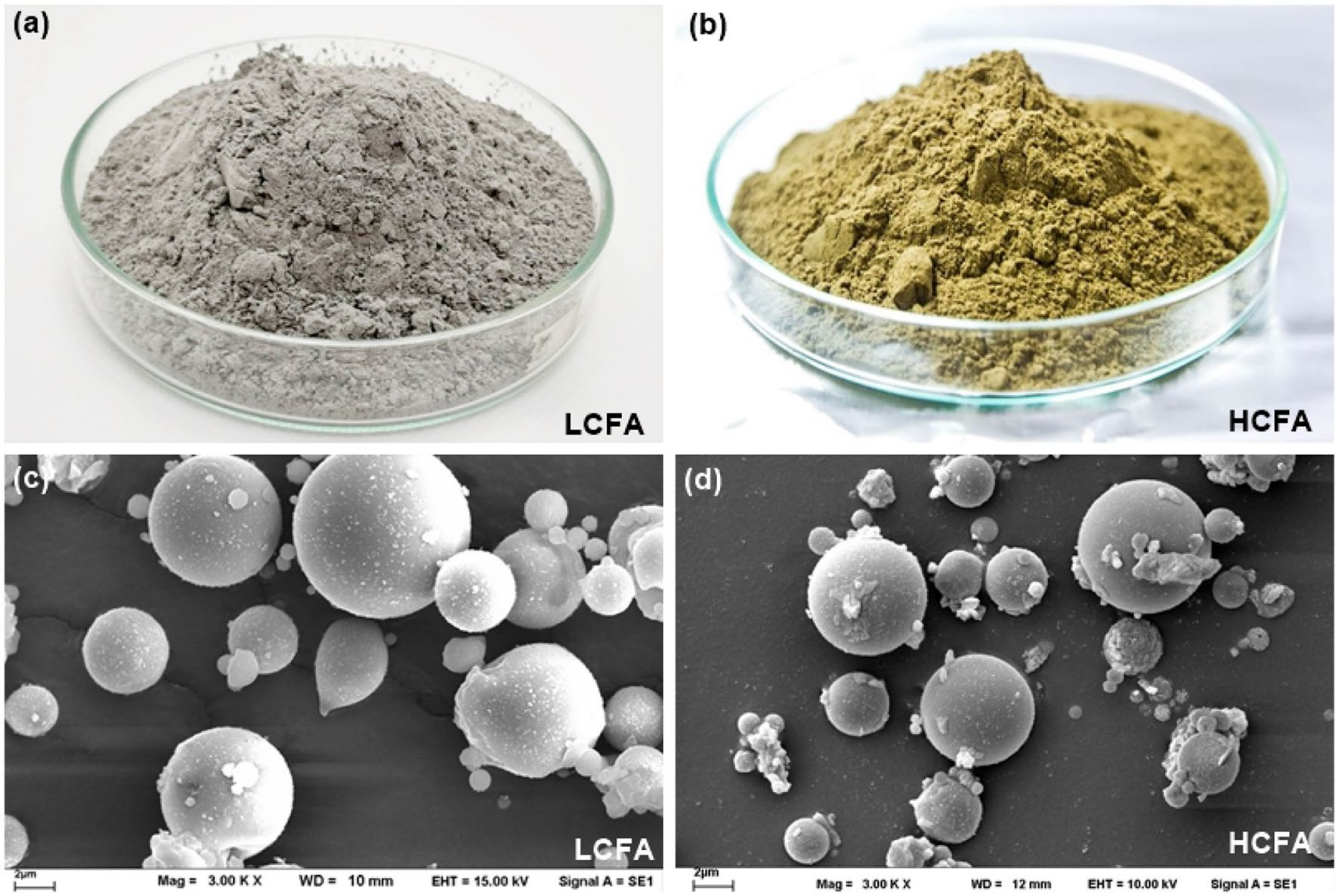
Colors and microstructures of the fly ashes.

Low-calcium fly ash (LCFA) from the BLCP power plant in Thailand was used as the aluminosilicate source for geopolymer synthesis. Bituminous coal was a feedstock of a pulverized coal combustion (PCC) process. The oxide composition of the fly ash measured by X-ray fluorescence (XRF) spectrometry is shown in Table 1. Quartz (SiO_2_) and aluminum oxide (Al_2_O_3_) were the main constituents. Iron oxide (Fe_2_O_3_) was a minor oxide, with 4.6% content, giving the ash its gray color. The sum of SiO_2_, Al_2_O_3_, and Fe_2_O_3_ was greater than 70%, as required for Class F according to ASTM C618-23^7^. The CaO content of this fly ash was 3.6%, which is reasonable because high-grade coal can reduce fuel retention time in the combustion chamber and result in a relatively dilute concentration of CaO residue^32^. The median particle size (D50) of the fly ash was 13.1 μm. High-calcium fly ash (HCFA) from the lignite PCC process of the Mae Moh power plant complied with Class C coal ash specifications and was used for comparison. The minimum combined content of SiO_2_, Al_2_O_3_, and Fe_2_O_3_ of 50% was detected for the Class C fly ash, with a high calcium content of 31.0 wt%. The median particle size (D50) of the Class C fly ash was 19.0 µm. A high Fe_2_O_3_ content of 15.5% resulted in a brownish color. The colors and microstructures of the LCFA and HCFA powders are shown in Fig. 1. The particles of both fly ashes had spherical shapes due to their rapid solidification during the suspension in the flue gas^33^.

**Table 1:** Chemical compositions of fly ashes and calcium additives.

Oxide compositions	LCFA (%)	HCFA (%)	Lime (%)	WC (%)	OPC (%)	CCR (%)
SiO_2_	67.4	25.9		17.1	20.9	4.7
Al_2_O_3_	20.1	13.7		14.9	4.8	2.5
Fe_2_O_3_	4.6	15.5		2.9	3.4	0.7
CaO	3.6	31.0	> 95*	60.7	65.4	89.6*
SO_3_	0.4	7.3		3.5	2.7	0.9
K_2_O	1.2	1.9		0.6	0.4	–
MgO	0.5	2.2		–	1.3	1.7
Na_2_O	0.4	1.4		–	–	–
Others	1.8	1.1		0.3	1.1	–

*Calcium forms in lime (CaCO_3_) and CCR (Ca(OH)_2_).

Four types of calcium additives were used for LCFA replacement to improve the geopolymer properties. These calcium additives consisted of WC slurry in powder form, CCR, ordinary Portland cement (OPC), and limestone powder. WC was supplied by a local concrete recycling company, and the concrete washing slurry was dried and sieved through a 100-mesh sieve (0.149 mm openings). CCR was obtained from a local acetylene plant and dried and ground into fine particles using a high-speed grinder. The ground CCR was also sieved through a 100-mesh sieve. Limestone powder and OPC were obtained from local suppliers and sieved through a 100-mesh sieve to remove the agglomerated particles. The chemical compositions of the additives are presented in Table 1. Median particle sizes of lime stone, WC, OPC and CCR were 39.8, 48.1, 14.6 and 42.3 μm, respectively. The fine river sand was mixed with geopolymer pastes to prepare the geopolymer mortar. The sand had a specific gravity of 2.7 and a fineness modulus of 1.9.

**Figure 2:**
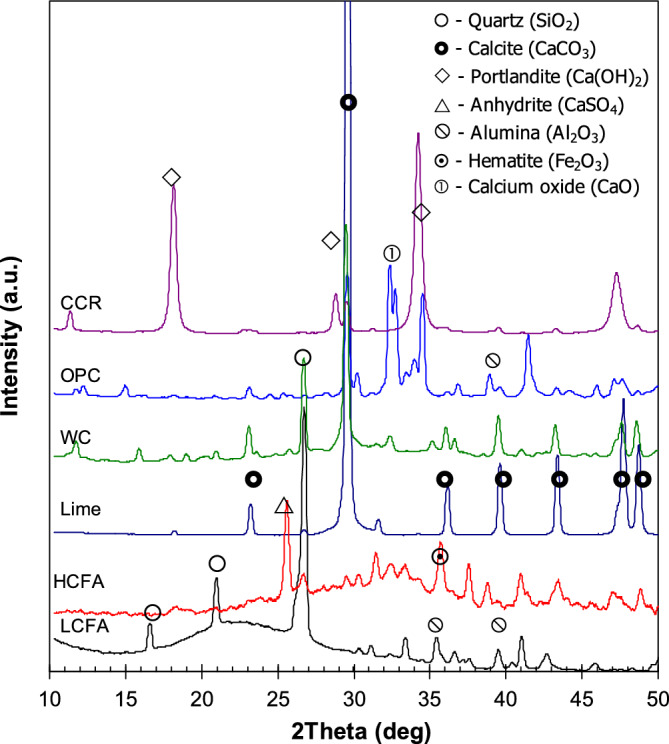
XRD patterns of the fly ashes and additives.

The XRD patterns of the fly ashes and additives are shown in Fig. 2. Peaks for SiO_2_, Al_2_O_3_, Fe_2_O_3_ and CaO can be identified. Although some sharp peaks for the crystalline structures were observed, broad peaks at 15–30° 2θ (for LCFA) and 25–40° 2θ (for HCFA) indicated the predominantly amorphous phases of the fly ashes. The pattern of LCFA showed a left-skewed distribution, indicating a high SiO_2_ content in the fly ash. In comparison, HCFA showed a right-skewed distribution, indicating its high CaO content. Fe_2_O_3_ was also detected in HCFA, according to the XRF results. Calcite, the cubic crystal structure of calcium carbonate (CaCO_3_), was a major component in limestone, whereas the portlandite or calcium hydroxide (Ca(OH)_2_) was significant in CCR. WC had a low peak height compared with OPC owing to the OPC hydration reaction.

Sodium silicate (Na_2_SiO_3_) solution and 10 M sodium hydroxide solution (NaOH) were used as the alkaline activators. The Na_2_SiO_3_ solution comprised soluble SiO_2_ (30 wt%), Na_2_O (9 wt%), and water (61 wt%). Concentrated HCl (37 wt%, 12 M) was diluted with water to obtain a 3% hydrochloric acid (3% HCl) for the durability study. Silver nitrate (AgNO_3_, AR grade) was dissolved in deionized water to prepare 0.1 M AgNO_3_ solution.

### Preparation of geopolymers

Calcium additives were used to replace the LCFA at 20% by weight, which was recommended for the evaluation of the strength activity index^34,35^. A Na_2_SiO_3_–NaOH ratio of 2 was employed for the alkaline solutions. A high amount of Na_2_SiO_3_ was used to supply the soluble SiO_2_ in the mixture. The alkaline solution-to-solid particles ratio of 0.65 was selected to obtain the fluid paste. For comparison, HCFA was also prepared.

The preparation of the control mix (G_LCFA) was established as follows: 60 g of fly ash was first mixed with 13.3 g of 10 M NaOH solution, and then 26.7 g of Na_2_SiO_3_ was added and mixed to obtain the homogeneous paste. For other samples, the fly ash and calcium additive were thoroughly dry mixed in a plastic mixing pan according to the mix proportions in Table 2. Then, the 10 M NaOH and Na_2_SiO_3_ were sequentially added and mixed until a uniform mixture was obtained. It should be noted that, for CCR, flash setting of the paste occurred when Na_2_SiO_3_ was added, owing to the high content of reactive calcium in CCR. Therefore, only 5% CCR was used to replace the LCFA and provide sufficient time for sample molding.

**Table 2:** Mix proportions of the geopolymers (based on 100 g of solid particles).

Samples	Fly ash (g)		Additives (g)	Na_2_SiO_3_ (g)	10 M NaOH (g)	Type of calcium additives
	LCFA	HCFA				
G_LCFA	60	–	–	26.7	13.3	Control
G_HCFA		60	–	26.7	13.3	Comparison
G_Lime	48	–	12	26.7	13.3	Limestone
G_WC	48	–	12	26.7	13.3	Waste cement
G_OPC	48	–	12	26.7	13.3	Ordinary Portland cement
G_CCR	57	–	3	26.7	13.3	Calcium carbide residue

The paste was poured into small silicone mold with the size of 3 × 3 × 2 cm^3^ and cured at 45 °C for 48 h^11^. The hardened pastes ware used for the microstructural and chemical analyses. Low-temperature curing was employed since outdoor heat exposure and power plant waste heat are available for heat curing of geopolymers in mass production^36^. The hardened geopolymers were demolded and kept in zip-lock bags. To mix mortar for strength tests, sand was used as fine aggregate with a sand-to-solid particle ratio of 1. Sand was first mixed with dry particles, and 10 M NaOH was added. The sample was thoroughly mixed before the addition of Na_2_SiO_3_. The fresh mortar was cast into 5-cm cube molds according to the ASTM C109/C109M-20^37^ and the same heat-curing treatment was applied.

### Testing and characterization

The structural properties of fly ashes, additives, and hardened geopolymer pastes were studied by X-ray diffraction (XRD). The pastes were also subjected to thermogravimetric analysis (TGA). The hardened pastes were ground and then passed a 100-mesh sieve (0.149 mm opening) and used for XRD and TGA analyses. In addition, element mapping at the microstructural level of the geopolymer surface was performed by scanning electron microscopy combined with energy-dispersive X-ray spectroscopy (SEM/EDX). Small piece of paste was used for SEM/EDX analysis.

For geopolymer mortars, there were two curing conditions, namely room temperature (RT) curing and 3% HCl curing for durability study. According to ACI 201.2R-01 *Guide to durable concrete*^38^, hydrochloric acid (HCl) is a common test for deterioration of concrete due to the rapid rate of attack at ambient temperature. Immersion of mortars in 3% HCl represented the aggressive environmental conditions to concrete structure. Compressive strength was tested after 90 days. The mortar cubes were sprayed with 0.1 M AgNO_3_ solution after failure under the compressive strength test to observe the chloride diffusion from acid into the specimens. The results were subjected to an analysis of variance, and *p* < 0.05 was considered significant.

## Results and discussion

### Phase identification of geopolymer pastes

**Figure 3:**
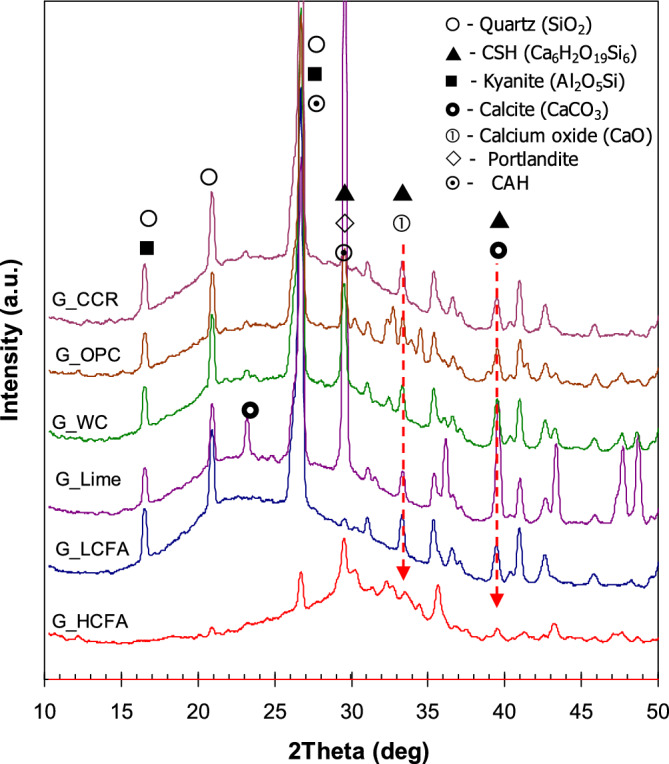
XRD patterns of the geopolymer pastes.

The XRD patterns of hardened geopolymer pastes are shown in Fig. 3. The main chemical compounds of the geopolymers were identified based on XRF results and the expected reaction products. Peaks were detected in XRD patterns consisting of unreacted phases from fly ash and calcium additives i.e. quartz (SiO_2_), calcite (CaCO_3_), calcium oxide (CaO) and trace oxide minerals. In addition, the reaction products in the geopolymer were identified on XRD patterns including an aluminosilicate compound (kyanite), CSH and portlandite (Ca(OH)_2_). These products were formed when fly ash reacted with alkaline solutions under heat activation. Due to the high calcium content in the geopolymer mixture, CSH was also detected on XRD patterns. The CSH was originated from the reaction between calcium compounds, which were available in both the HCFA and calcium additive, and alkaline solutions. CSH was detected at approximately 29, 34 and 39°2θ, and kyanite was detected at 26°2θ. A small peak of calcium aluminate hydrate (CAH) was detected at 26 and 29°2θ, which were at the same peak locations as kyanite and CSH. The broad peaks of all mixtures were noticeable implying the presence of amorphous phases of geopolymers. It has been reported that the curing temperature can affect the formation of product. CSH is the main product in RT curing geopolymer, whereas aluminosilicate compound is formed at high temperature curing exceeding 35 °C^39^.

### Compressive strength of geopolymer mortars

**Figure 4:**
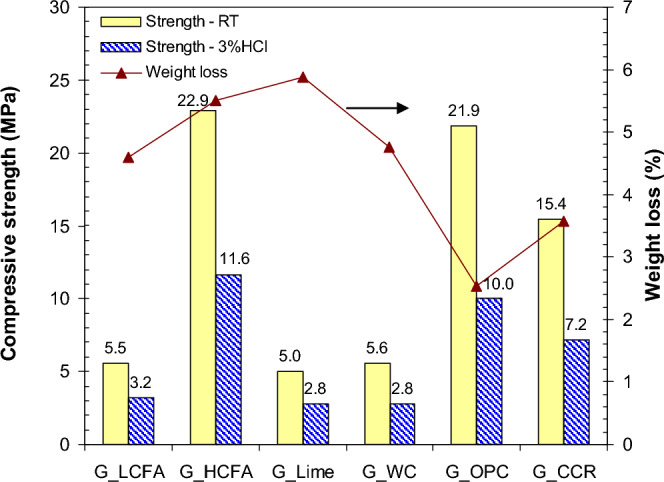
Compressive strengths and weight loss of the geopolymer mortars.

The compressive strength tests were performed on the geopolymer mortars after 90 days of curing at RT or in 3% HCl solution, representing the highly acidic environment. The weight loss of geopolymers under 3% HCl conditions was calculated based on the initial weights of the geopolymers. Figure 4 shows the results of the compressive strength tests and weight loss calculations. For RT curing, the compressive strength of the HCFA geopolymer (22.9 MPa) was significantly higher than that of the LCFA geopolymer (5.5 MPa) because of the CSH formation. Additions of limestone powder and WC did not significantly affect the strength of the geopolymers, indicating the low reactivity of the calcium compounds.

WC had a low reactivity since it was the product from the hydration reaction of OPC. The incorporation of WC in geopolymer resulted in numerous crystalline phases and unreacted substances leading to a relatively low strength geopolymer^31^. In case of limestone powder, it has been reported that use of limestone powder at 20% in concrete mixture reduced the compressive strength as the result of a “clinker dilution effect” from replacing a part of cement by the same quantity of limestone. It was thus recommended the incorporation of limestone powder at 10% for the contribution of strength due to filler and nucleation effects^40,41^.

In comparison, the addition of OPC (20% LCFA replacement) and CCR (5% LCFA replacement) resulted in noticeable increases in the compressive strength of LCFA geopolymers, in the range of 15–22 MPa. Apart from the hydration reaction of OPC, the pozzolanic reaction occurred in the presence of calcium compounds, an alkaline solution, and an aluminosilicate source from LCFA. For the CCR additive, active Ca(OH)_2_ directly reacted with the Na_2_SiO_3_ solution, resulting in a fast setting time. Notably, for the trial mix with CCR and 10 M NaOH, a slurry paste was obtained with no sign of flash setting. Hence, dissolved silica from the Na_2_SiO_3_ solution played an important role in the hardening of the CCR mixture.

For the immersion in 3% HCl solution, all samples showed a significant decrease in compressive strength, with approximately 50% reduction. A curing temperature of 45 °C for 48 days appeared to be inadequate to obtain a strong geopolymer matrix. The outdoor heat exposure of the HCFA geopolymer was recommended for 5 days to obtain a strong matrix with high strength gain^13^. The high heat during curing activated the geopolymerization reaction earlier, resulting in the formation of more durable aluminosilicate compounds with higher acid resistance^42^. However, the aluminosilicate network in the geopolymer can also be destroyed by H^+^ (from HCl ionization), leading to a broken oxy-aluminum bridge and deterioration of the geopolymer. The use of calcium additives resulted in free calcium compounds in the geopolymer, leading to low acid resistance. These compounds can easily be attacked by acid solution and leach out from the composites, resulting in pores and cavities^43^. It has been reported that the decalcifications of portlandite (Ca(OH)_2_) and CSH lead to reductions in the cementing properties such as paste cohesion, binding, strength, and durability^44^.

The strength reduction and significant weight loss were observed after 3% HCl immersion. This was caused by the leaching of calcium and resulted in physical and chemical damage. In the case of the G_OPC sample, the weight loss was high since spalling occurred at the corners of the cubes. The weight losses of G_OPC and G_CCR geopolymers were notably lower than those of the HCFA and LCFA geopolymers, indicating the enhanced strength of the matrix with low permeability. However, the CSH product from this geopolymeric system was more sustainable than that from cement-based materials. Previous research has shown that the cement-based materials started to dissolve in 3% H_2_SO_4_ solution within the first week of immersion^45^.

**Figure 5:**
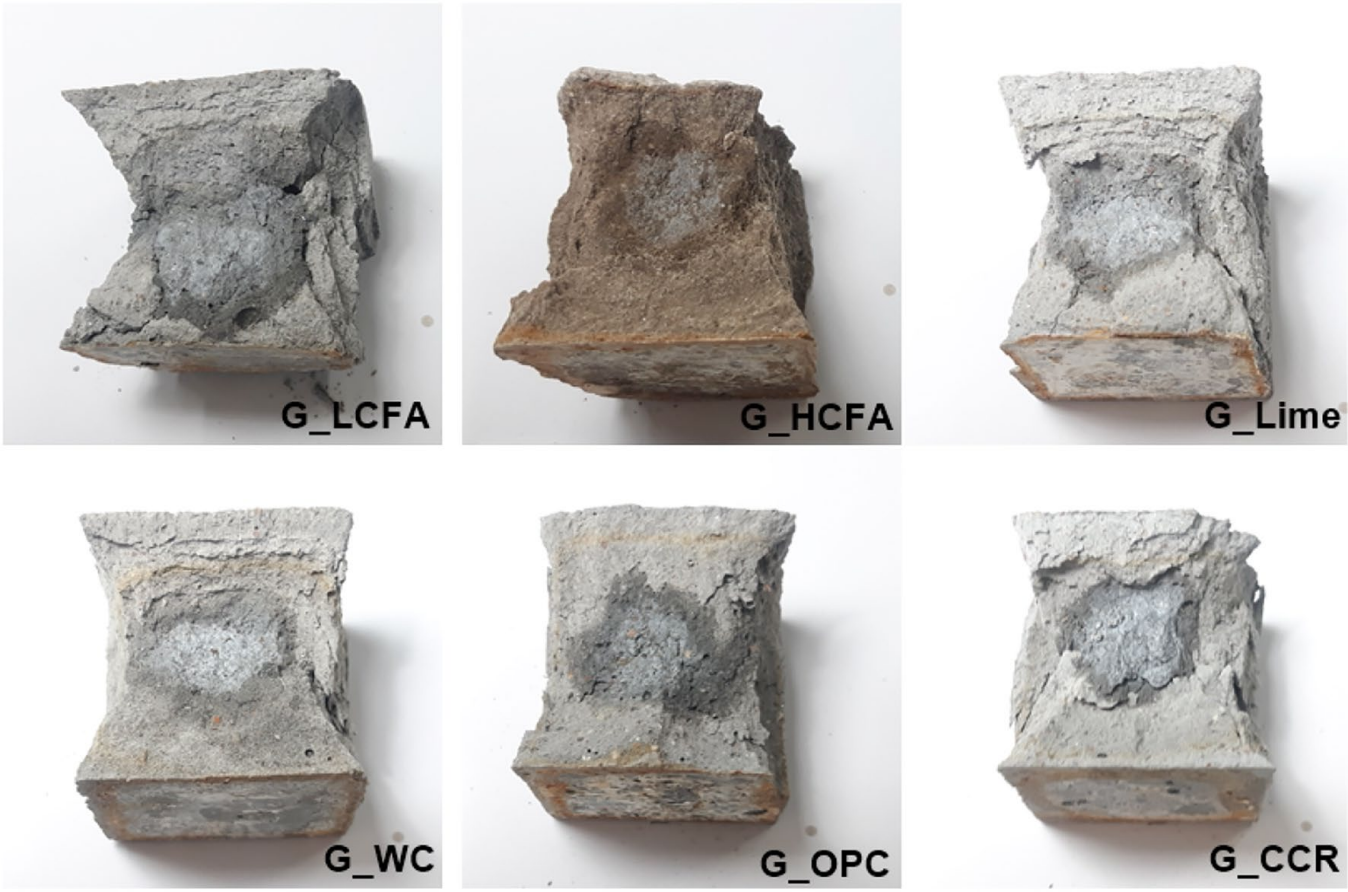
Precipitates of AgCl on broken surfaces of the geopolymers.

The mortar cubes were sprayed with 0.1 M AgNO_3_ solution after failure in the compressive strength tests to observe the chloride diffusion from the acid solution into the specimens. The presence of chloride ions (Cl^−^) in the geopolymer matrix indicated the chloride diffusion, and the results are shown in Fig. 5. The Cl^−^ ions came from the deposition of a strong acid (3% HCl) and reacted with AgNO_3_ solution, resulting in the white precipitate of AgCl. The reaction is shown in Eq. (1).

1 $${\text{Cl}}^{ - } \left( {{\text{aq}}} \right) + {\text{AgNO}}_{{3}} \left( {{\text{aq}}} \right) \to {\text{AgCl}}\left( {\text{s}} \right) + {\text{NO}}_{{3}}^{ - } \left( {{\text{aq}}} \right)$$

The Cl^−^ ions diffused into all geopolymer matrixes, as indicated by the white precipitate. A large quantity of white precipitate was formed in the LCFA geopolymers blended with limestone powder (G_Lime) and WC (G_WC). This may be due to the increased generation of pores and cavities during 3% HCl immersion, leading to more chloride penetration.

Since the calcium waste additive plays the important role on the strength of geopolymer, therefore an application of these geolymers is carefully considered. For the low strength geopolymer, non-load bearing application was chosen, for instance, a non-load bearing walls, ceiling board, or porous geopolymer. It has been reported that porous geopolymer can capture the CO_2_ gas owning to the presence of a tetrahedrally coordinated lattice of Al or Si atoms from fly ash, and Na^+^ from alkaline solutions^46^. The additional presence of Ca^2+^ ion from calcium waste could help to promote the CO_2_ capture from the flue gas. The porous flu ash geopolymer has a CO_2_ uptake slightly lower than that of the zeolite, therefore, it is a promising absorbent material with environmentally friendly and alternatively waste utilization^14^.

### Microstructural study

**Figure 6:**
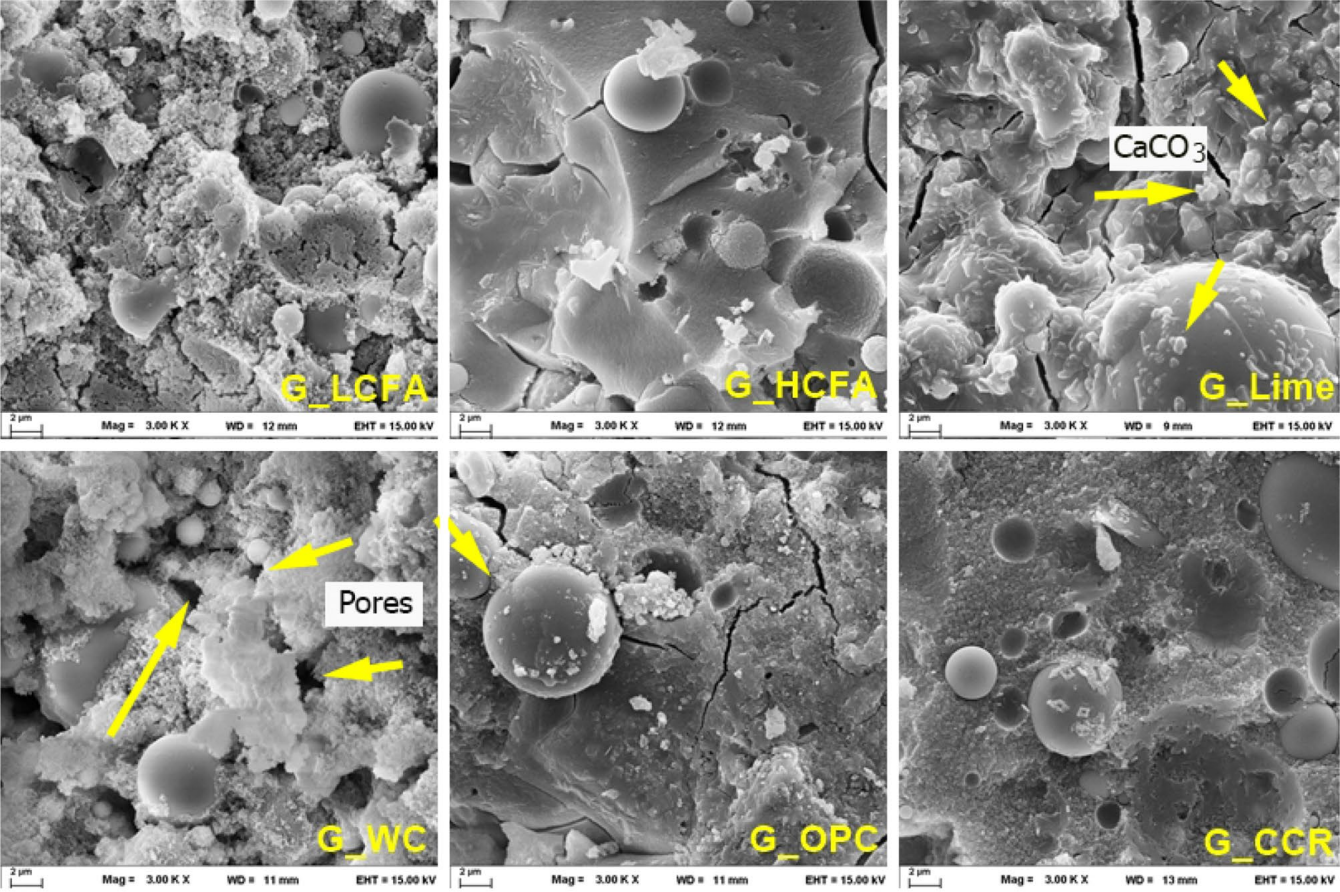
Microstructures of the geopolymers.

The microstructures of geopolymer pastes observed by SEM are shown in Fig. 6. Unreacted and partially reacted fly ash and continuous masses of geopolymer matrixes were common features. Fly ash is composed of crystalline phases, particularly quartz in the internal regions and a glassy phase of silica and alumina at the surface layer^47^. This glassy phase plays an important role in geopolymer gel formation, resulting from the high solubility in the alkaline solution. The matrix structure of the HCFA geopolymer was dense and significantly different from that of the LCFA geopolymer. The loose matrix with a small amount of hardened geopolymer gel was observed in the LCFA geopolymer. This affected the density and reduced the strength of the geopolymer. The results suggested that an increase in heat-curing time can result in more reaction products on the fly ash particles.

For the G_Lime sample, cube calcite (CaCO_3_) was observed in an agglomerated form, adhered to the fly ash particles. A porous matrix was also observed in the G_WC sample, indicating the low reactivity of the WC powder; therefore, it acted as a filler in the composite. For the OPC and CCR calcium additives, a dense matrix was observed in both cases, leading to high compressive strengths.

**Figure 7:**
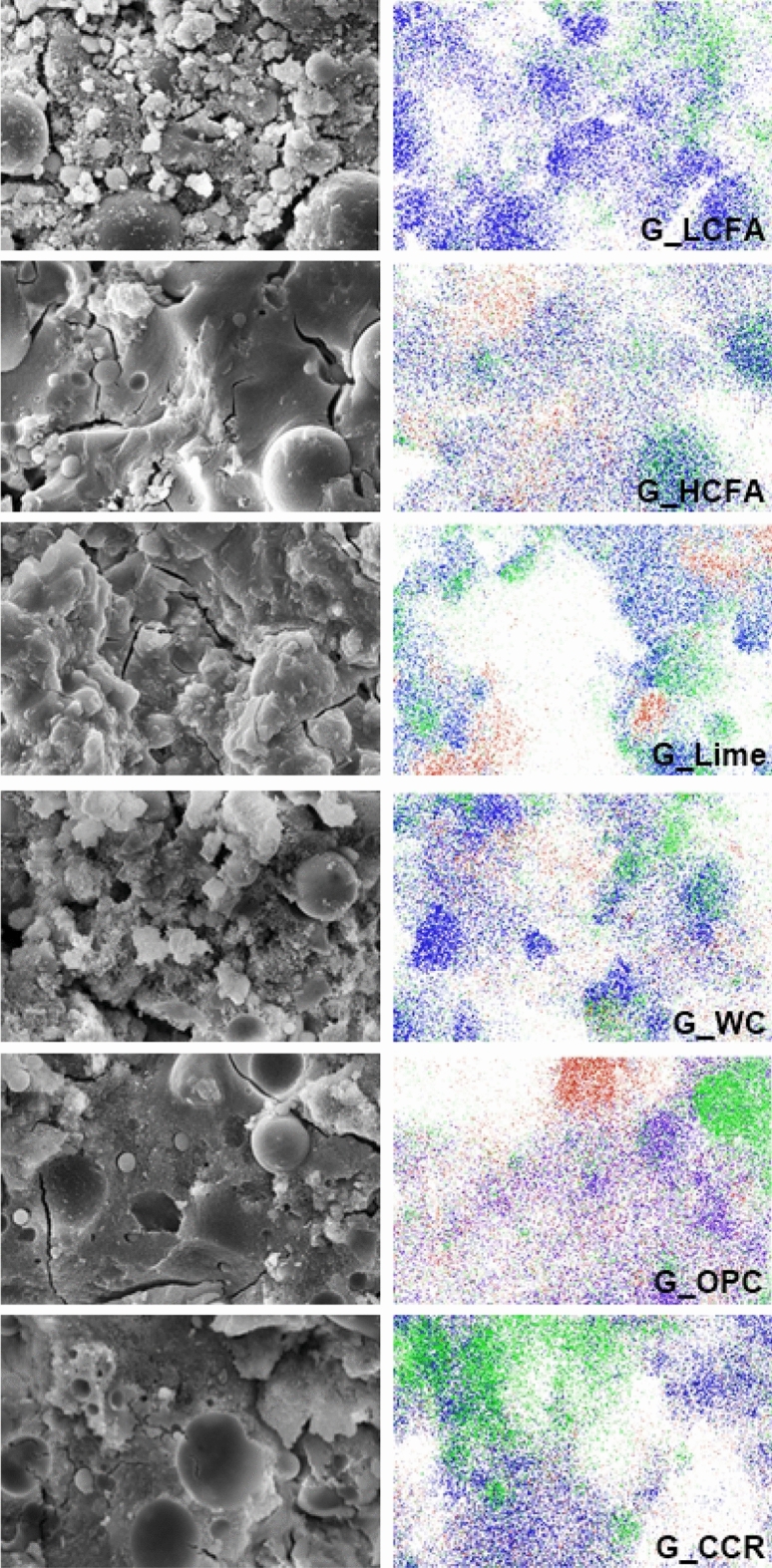
Elemental mapping of common elements in geopolymer matrixes (blue = Si, green = Al, red = Ca).

Elemental mapping at the microstructural level of the geopolymer surfaces was conducted by SEM/EDX. Si, Al, and Ca elements were investigated and color was applied. Elemental maps of the three elements in the geopolymers matrixes at 5000 × magnification are shown in Fig. 7. The mapping area was 30 μm × 40 μm, in which the blue color is Si, the green color is Al, and the red color is Ca.

For G_LCFA, Si and Al atoms were distributed throughout the matrix, and Si atoms were also concentrated around the fly ash particles. Since amorphous SiO_2_ and Al_2_O_3_ generally existed at the surface^47^, these atoms easily leached out from the surface in alkaline solutions. G_HCFA exhibited a distribution of Ca, Si, and Al atoms, demonstrating the formation of CSH and CASH. Si and Al were also found in the fly ash particles, implying that Ca reacted more with Si from the alkaline solution than Si and Al from the fly ash. The same phenomenon was revealed in the G_OPC and G_CCR samples. In particular, Ca atoms were distributed throughout the matrix of G_OPC. The activation energy (E_a_) of the CSH compound is reported to be ~ 30 kJ/mol^48^ and that of the aluminosilicate compound is ~ 60 kJ/mol^49^. The activation energy of a chemical reaction is related to its reaction rate and chemical kinetics. Significantly, a lower activation energy results in a faster chemical reaction since the molecules can complete the reaction once they have overcome the activation energy barrier. With high activation energy, fewer molecules have enough energy to form the products and a larger energy input is required to initiate the reaction. Regarding the activation energy, CSH formed faster than aluminosilicate in the presence of active calcium additives and alkaline solution in the LCFA geopolymer system.

### Thermal analysis

**Figure 8:**
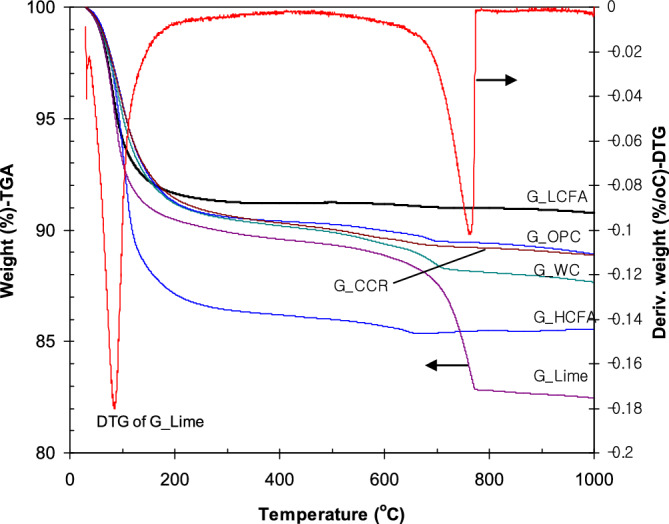
TGA/DTG results for the geopolymer pastes.

TGA and differential thermogravimetry (DTG) were performed on the geopolymer pastes, and the results are shown in Fig. 8. The TGA curve showed the percentage of weight remaining as a function of heating temperature under a nitrogen atmosphere, whereas the DTG curve presented the decomposition temperature of a specific volatile compound in the materials. The DTG curve of the G_Lime sample was selected as representative since the DTG curves of all samples appeared at similar temperatures.

The geopolymers exhibited weight changes at approximately 100 and 750 °C, which corresponded to water evaporation, and decomposition of CaCO_3_ into CaO and CO_2_^13^. The DTG peak at 100 °C was related to the evaporation of water from the NaOH and Na_2_SiO_3_ solutions and to a pore solution in the pastes. High weight loss was observed in the temperature range of 700–800 °C, which was due to the decomposition of CaCO_3_. This indicated that CaCO_3_ was the main compound in the geopolymers, particularly in G_Lime. G_LCFA had a lower weight loss of 8% because of the formation of Si–O–Al bonding and high thermal resistance. G_HCFA had higher weight loss than the LCFA geopolymer owing to the existence of free calcium in the fly ash. Heat curing and alkaline solutions converted the reactive calcium additives in the LCFA geopolymer into more thermally stable calcium compounds (i.e., CSH), as lower weight losses of approximately 12% were detected in G_OPC and G_CCR compared with those of G_WC and G_Lime. Weight loss was high for the limestone additive, resulting in a remaining weight of 82%. However, this remaining weight was significantly higher than that of the conventional OPC pastes (W/C ratios of 0.30–0.33); their remaining weight was approximately 65% of the initial weight^50,51^.

The differences in CaCO_3_ decomposition among all samples at 750 °C were visible in the TGA curves. The different weight losses (or weight change) between the peaks at 100 °C (first peak) and the peaks at 750 °C (second peak) were calculated, as shown in Table 3. The weight changes of G_HCFA and G_OPC at 750 °C were approximately 0.8%; however, the total weight losses were dramatically different. The weight loss of G_HCFA occurred mainly at 100 °C, owing to the evaporation of water and the excess alkaline solution used in the mixture. The CaCO_3_ decomposition was also low in G_CCR (1.0%). These three samples had high compressive strength values. High CaCO_3_ decomposition was observed in G_WC (2.0%) and G_Lime (6.8%) as shown in Table 3. The high decomposition of CaCO_3_ conformed to the low strength of G_WC and G_Lime geopolymers. Therefore, the CaCO_3_ decomposition at 750 °C of the composites (weight loss) can be inversely related to the compressive strength results of Ca/LCFA and HCFA geopolymers. The low weight loss of CaCO_3_ corresponded to the high compressive strengths obtained as a result of the calcium additive reactivity and the formation of thermally stable CSH. From the results, CCR was the most favorable calcium additive for LCFA. For the utilization of CCR in geopolymer systems, the amount of alkaline solution should be adjusted for the workability of the mixture. Similarly, a higher amount of NaOH solution than Na_2_SiO_3_ solution has been recommended to avoid the fast setting of the mixture^21^. However, high Ca(OH)_2_ formation may increase the plastic shrinkage of alkali-activated concrete.

**Table 3:** CaCO_3_ decomposition at 750 °C according to the difference in weight loss.

Samples	Residue at 100 °C (%)	Residue at 750 °C (%)	Weight change (%)	Compressive strength (MPa)
G_HCFA	86.196	85.341	0.855	22.9
G_Lime	89.549	82.705	6.844	5.0
G_WC	90.124	88.089	2.035	5.6
G_OPC	90.339	89.479	0.860	21.9
G_CCR	90.283	89.219	1.064	15.4

## Conclusions

Four calcium additives were blended with LCFA for geopolymer production. An HCFA geopolymer was also tested for comparison. The reactivity of calcium additives played an important role in the formation of CSH and changes in strength. In the calcium–aluminosilicate–alkali system, CSH was easily formed compared with Al–O–Si bonding. A maximum strength of 22.9 MPa was obtained from HCFA geopolymers cured at 45 °C, which is consistent with the application of outdoor heat exposure (solar energy). Immersion in 3% HCl solution had deleterious effects on the strengths of the geopolymers. Higher curing temperatures or longer heat-curing times resulted in more durable aluminosilicate compounds. OPC and CCR were the favorable reactive sources of calcium for blending with LCFA. Low thermal weight changes with higher strength gains were achieved. Low remaining CaCO_3_ contents at 750 °C indicated the formation of thermally stable CSH and was associated with high compressive strength in the Ca/LCFA geopolymers. CCR is an effective calcium additive for LCFA, which is advantageous considering it can be derived from industrial waste. For the application of CCR in geopolymer systems, it is necessary to adjust the amount of alkaline solution to control the workability of the mixture.

## Data Availability

The datasets used and/or analyzed during the current study are available from the corresponding author on reasonable request.
